# FRAILTY IN INTERMEDIATE CARE FACILITIES THROUGH LIFE (LONG-TERM CARE INFORMATION SYSTEM FOR EVIDENCE) IN JAPAN

**DOI:** 10.1093/geroni/igae098.2761

**Published:** 2024-12-31

**Authors:** Tomomichi Sakai, Renuka Visvanathan, Agathe Daria Jadczak, Hiroyuki Umegaki

**Affiliations:** Nagoya University Graduate School of Medicine, Nisshin, Aichi, Japan; University of Adelaide, Adelaide, South Australia, Australia; University of Adelaide, Adelaide, South Australia, Australia; Nagoya University, Nagoya, Aichi, Japan

## Abstract

The Long-term care Information system For Evidence (LIFE) was introduced to improve care for older adults based on scientific evidence in 2021 within the Japanese long-term care insurance framework. The aim of this study was to reveal the prevalence of frailty among residents in intermediate care facilities and identify factors associated with the frailty using data collected for the emerging system. This retrospective and cross-sectional study included residents aged 65 years or above who had complete data for assessing frailty. Data was collected between April 2021 and November 2023 at 4 intermediate care facilities. Residents were classified as non-frail, frail, or most-frail using the FRAIL-NH scale by fitting variables in the LIFE data that were most appropriate for each component of the FRAIL-NH scale. Multivariable logistic regression analysis was used to explore factors associated with being most-frail using variables covering broad areas such as sociodemographic information, nutrition, oral function, and cognition. 907 residents were included for analysis; their mean age was 85.9 (standard deviation 7.2) years, and 63.7% were female. Among them, 5.7%, 33.8%, and 60.4% were categorized as non-frail, frail, and most-frail. The necessity of a dysphagia diet, easily choking, and dementia status and symptoms were shown to be significantly associated with being most-frail using the reference of not being most-frail. The current results indicate the potential for frailty assessment using data that recently started being collected routinely across Japan. Future studies of the predictive role of frailty against prognosis should promote care delivery stratified by frailty assessment.

